# Protein Supplement Tolerability and Patient Satisfaction after Bariatric Surgery

**DOI:** 10.1007/s11695-024-07462-4

**Published:** 2024-09-07

**Authors:** Cornelia Lianda H. Luijpers, Malou A. H. Nuijten, Evi J. Groenhuijzen, Lilian L. van Hogezand, Valerie M. Monpellier, Thijs M. H. Eijsvogels, Maria T. E. Hopman

**Affiliations:** 1 Department of Medical BioSciences (Route 928), Radboud University Medical Center, P.O. box 1901, 6500 HB, Nijmegen, The Netherlands; 2 Nederlandse Obesitas Kliniek (Dutch Obesity Clinic), Amersfoortseweg 43, 3712 BA Huis Ter Heide, The Netherlands; 3 Department of Surgery, St. Antonius Hospital, Koekoekslaan 1, 3435 CM Nieuwegein, The Netherlands

**Keywords:** Roux-en-Y Gastric Bypass, Sleeve Gastrectomy, Dietary Complaints, Protein Intake

## Abstract

**Purpose:**

Disproportional fat-free mass loss often occurs post-bariatric surgery, partly due to insufficient protein intake during the post-surgery recovery phase. We compared five protein-enhancing strategies (PES) on patient tolerability, satisfaction and protein intake.

**Materials and Methods:**

Ninety-four participants, scheduled for bariatric surgery, were enrolled and allocated to either of the following: (1) whey powder, (2) hydrolysed collagen powder, (3) plant-based powder, (4) protein-rich products, (5) protein gel, or control. PES groups were instructed to add 30 g of powder or 2 gels or protein products to their diet. Patient satisfaction and tolerability were evaluated with questionnaires. Dietary intake was assessed prior to and during PES use.

**Results:**

Seven patients dropped out (i.e. loss of contact, personal reasons or post-surgery complications) yielding an analytical cohort of 87 participants. The majority of patients (61%) did not experience dietary complaints from PES and could use PES ≥ 5 days of the week. PES non-usage was mainly related to taste dislike (58%). Hydrolysed collagen scored highest on tolerability and satisfaction: 86% of the participants could use HC ≥ 5 days and 71% were satisfied with the product. PES increased protein intake from 54.7 ± 21.5 g/day to 64.7 ± 23.4 g/day during the intervention (*p* = 0.002), which differed from the control group (+ 10.1 ± 24.5 g/day vs. − 6.3 ± 23.8 g/day for controls, *p* = 0.019). Whey showed the highest increase, namely + 18.3 ± 16.3 g/day (*p* = 0.009).

**Conclusion:**

PES were tolerated by the majority of participants, and an improved protein intake with PES use was seen. However, the taste of the products could be improved to further enhance satisfaction and tolerability.

**Graphical Abstract:**

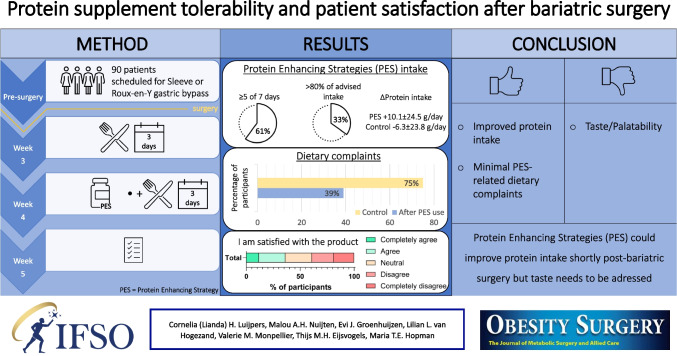

**Supplementary Information:**

The online version contains supplementary material available at 10.1007/s11695-024-07462-4.

## Introduction

Obesity has become a pandemic [1, 28], with an increased risk of other diseases like cardiometabolic disease and cancer [14, 18]. Metabolic and bariatric surgery (MBS) is effective in significantly reducing weight (~ 20–30% total weight loss) and associated health risks [13, 30, 32]. However, previous studies have shown a significant proportion of fat-free mass loss (FFM) with weight loss [9, 26]. Too much FFM loss can be harmful since FFM is crucial for bodily functions like bone health, insulin resistance and daily functioning [4, 15, 38].

Preservation of FFM by sufficient protein intake and resistance training should be a pillar in perioperative care programmes [38]. Guidelines recommend a daily protein intake of ≥ 60 g [25, 36, 37], which is challenging given the reduced gastric capacity and changes in taste, smell and digestion following MBS. Hence, protein-rich foods like meat and dairy products are less tolerated [10, 23, 34]. Studies found protein intakes of 29–45 g/day at 1 month post-surgery [12, 27]. Protein supplements or protein-rich products may be helpful to increase daily protein intake, but bariatric-induced changes in the digestive tract may affect tolerability of such products. Currently, tolerability of protein-enhancing strategies (PES) post-MBS remains unknown.

We assessed the effect of five different PES on (1) tolerability, (2) satisfaction and (3) total protein intake in patients after MBS. We hypothesised that PES could improve daily protein intake, with greater benefits for plant-based proteins, as better tolerability is expected due to the aversion to food with high animal protein content, and hydrolysed collagen due to the enhanced digestibility of hydrolysed protein [19, 23].

## Methods

### Study Population

Participants were recruited via the Dutch Obesity Clinic between September 2022 and August 2023. Patients scheduled for a primary Roux-en-Y gastric bypass or sleeve gastrectomy and participating in the perioperative care programme were eligible. Patients were excluded if they (1) had a language barrier, (2) were lactose intolerant or (3) could not use animal products due to (religious) beliefs. Ethical approval was obtained (file: #2022–13442), and all participants provided informed consent prior to assessments.

### Study Design

**Fig. 1:**
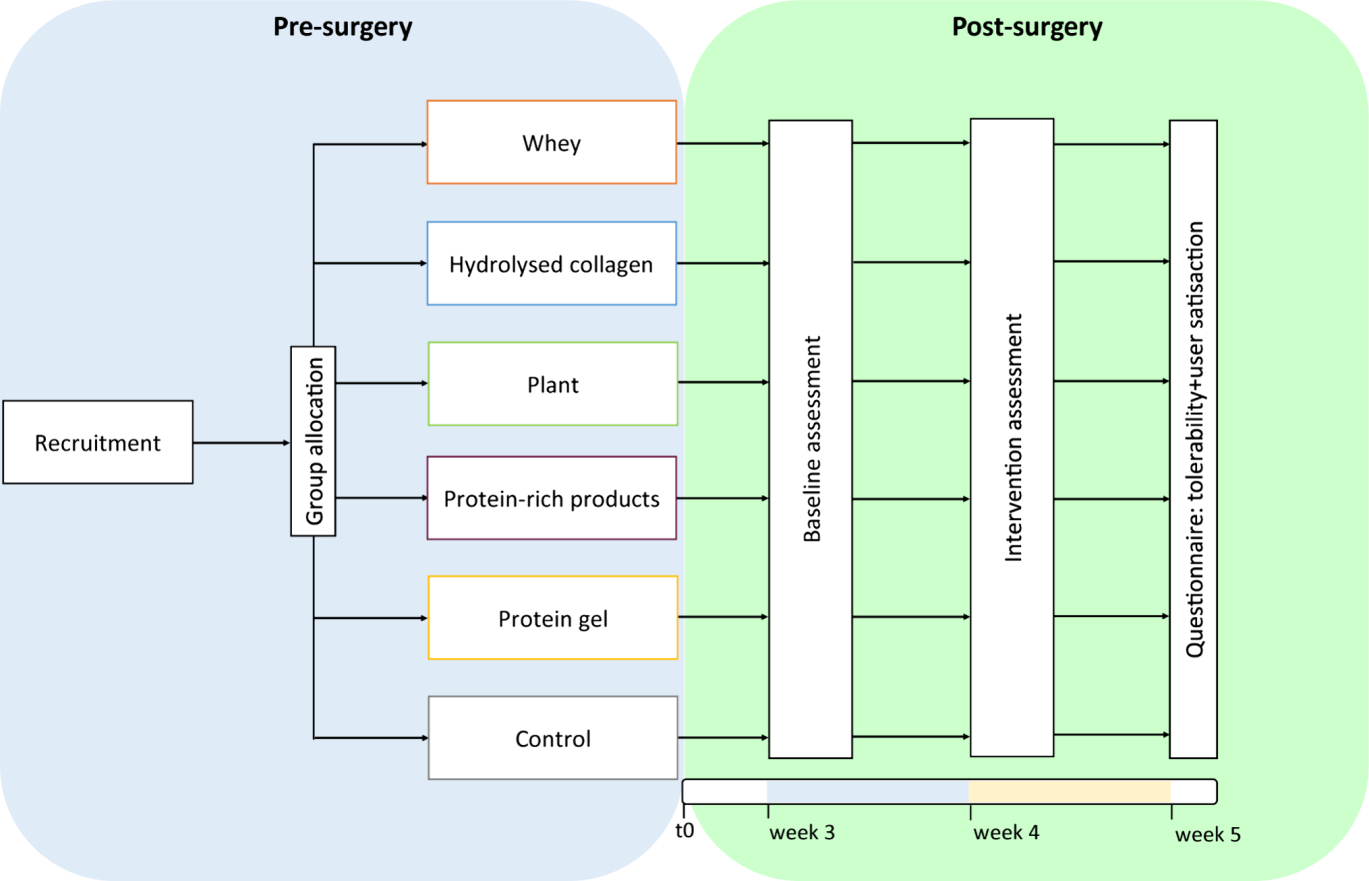
Overview of study design. Participants were recruited pre-surgery after which they were allocated to one of six study arms: 5 PES arms and 1 control group. At t0, participants underwent bariatric surgery. Baseline measurements were taken in the third week post-surgery, and in week 4, the intervention took place. After the fourth week, participants received the final digital questionnaire on tolerability and satisfaction with PES

Participants were allocated to one of six study arms. Dietary intake and dietary complaints were assessed on three days at baseline (i.e. 1 week pre-intervention) and during the intervention week. Participants in PES arms received protein supplements or products besides usual care, while the control group followed usual care. All participants received a digital questionnaire to assess tolerability and satisfaction within 1 week post-intervention (Fig. 1).

### Usual Care

All participants followed the usual care programme of the Dutch Obesity Clinic, which was previously described in detail [35]. In short, patients attend group sessions (*n* = 10 patients) to help them become self-sufficient in a healthy lifestyle. Advice was given on multiple lifestyle aspects, but no strict regimens were imposed. Regarding diet, patients were advised to eat three main meals and three smaller meals a day to allow sufficient nutrient intake and to consume protein at each meal and two or three dairy products daily.

### Protein-enhancing Strategies

Five consumer-available PES differing in protein source, processing technique and protein concentration were assessed (Table 1). PES included three protein powders (FortiFit Powder from Nutricia (whey), Peptan from Rousselot (hydrolysed collagen, HC) and Pea protein from New Care (plant)), consumer-ready protein-rich products from ProWell (PRP) and protein gel from Dutch Medical Food (protein gel). The aim was to add 30 g of powder or two products or gels to the daily diet during the intervention.

**Table 1:** Overview of protein-enhancing strategies used in the study

Study arm	Product	Supplier Headquarter location	Product type	Protein source Animal (A) or plant (P)		Protein	Advised daily intake	Effective protein intake
Whey	FortiFit Powder	Nutricia, Zoetermeer, the Netherlands	Powder *	A	Whey from cows milk + leucine	51.6 g per 100 g	30 g of powder	15.5 g
HC	Peptan	Rousselot, Son, the Netherlands	Powder	A	Hydrolysed collagen from pork rinds	90 g per 100 g	30 g of powder	27 g
Plant	Pea protein	New Care Supplements, Waalwijk, the Netherlands	Powder	P	Pea protein isolate	85 g per 100 g	30 g of powder	25.5 g
PRP	Sarcobox	ProWell, Lennik, Belgium	Consumer-ready products	A/P	Protein from milk/egg or soy/wheat/pea	9.3–16 g per product (Supplement 1)	2 products	19.8–31 g
Protein gel	ProtiMedic	Dutch Medical Food, Venlo, the Netherlands	Thick liquid	A	Whey from cows milk + bovine hydrolysed collagen	50 g per 100ml	2 sachets of 40 mL	40 g

*Enriched with minerals and vitamins

Participants received PES by mail with instructions including example recipes and day menus for incorporation of PES in their diet. Example recipes were created by the researchers in consultation with dieticians and PES providers. Participants were advised to use PES twice daily. If unable to reach the advised daily intake, participants were encouraged to find a tolerable amount.

### Measurements

#### Dietary Intake

Dietary intake was assessed at baseline and during intervention. Participants logged dietary intake in a validated mobile application (Traqq, Wageningen University, Netherlands) for 3 days each week [21]. Participants received five push notifications daily (at 6.00, 10.00, 14.00, 18.00 and 22.00 h) to log their intake. The reporting window was 1.5 h. Participants reported mealtime, consumed products (including PES) and their quantity.

Dietary logs were checked for completeness and correctness by an independent researcher and updated if needed. Subsequently, energy and macronutrient intake was calculated by a validated web-based platform (Compl-eat, Wageningen University, Netherlands) [22]. Nutritional values were determined per product and per day as absolute values (e.g. kcal, grams, millilitres) and relative to total daily energy intake (en%) by using the Dutch Food Composition Database (NEVO version 7.1) [2]. Data from dietary logs were included when intake was logged correctly for ≥ 2 days per week during baseline and intervention. A correct day was defined as data entry following ≥ 3 daily push notifications.

#### Questionnaires

Protein tolerability, the ability to use PES in the advised dosage without experiencing more dietary complaints than with habitual diet, was assessed. A short questionnaire was presented after each push notification from the mobile application assessing overall dietary complaints and PES use (Supplement 2). The taste, ease-of-use and overall satisfaction of PES were scored on a 5-point Likert scale. Overall dietary complaints were assessed in all groups, whereas PES-induced dietary complaints were collected in PES groups only (Supplement 3 + 4).

### Statistical Analysis

Statistical analyses were performed using SPSS 29 (IBM Corp., Armonk, NY, USA). All continuous variables were visually inspected and tested for normality with the Shapiro–Wilk test and displayed as mean ± standard deviation (SD) or median [interquartile range]. Categorical data was presented as count (percentage). Either parametric or non-parametric analyses were performed depending on normality of the data. Differences in baseline characteristics and dietary intake between groups were analysed with chi-square test, Kruskal–Wallis or ANOVA. Changes in macronutrient intake from baseline to intervention within groups were analysed with paired *t*-test or Wilcoxon signed rank test. Independent *t*-test was used to compare the protein intake of PES arms at baseline and intervention to control and ∆protein intake from PES to control. Univariate ANOVA was used for the effect of PES on protein intake when confounding for baseline protein intake. Statistical significance was assumed at *p* < 0.05.

## Results

**Fig. 2:**
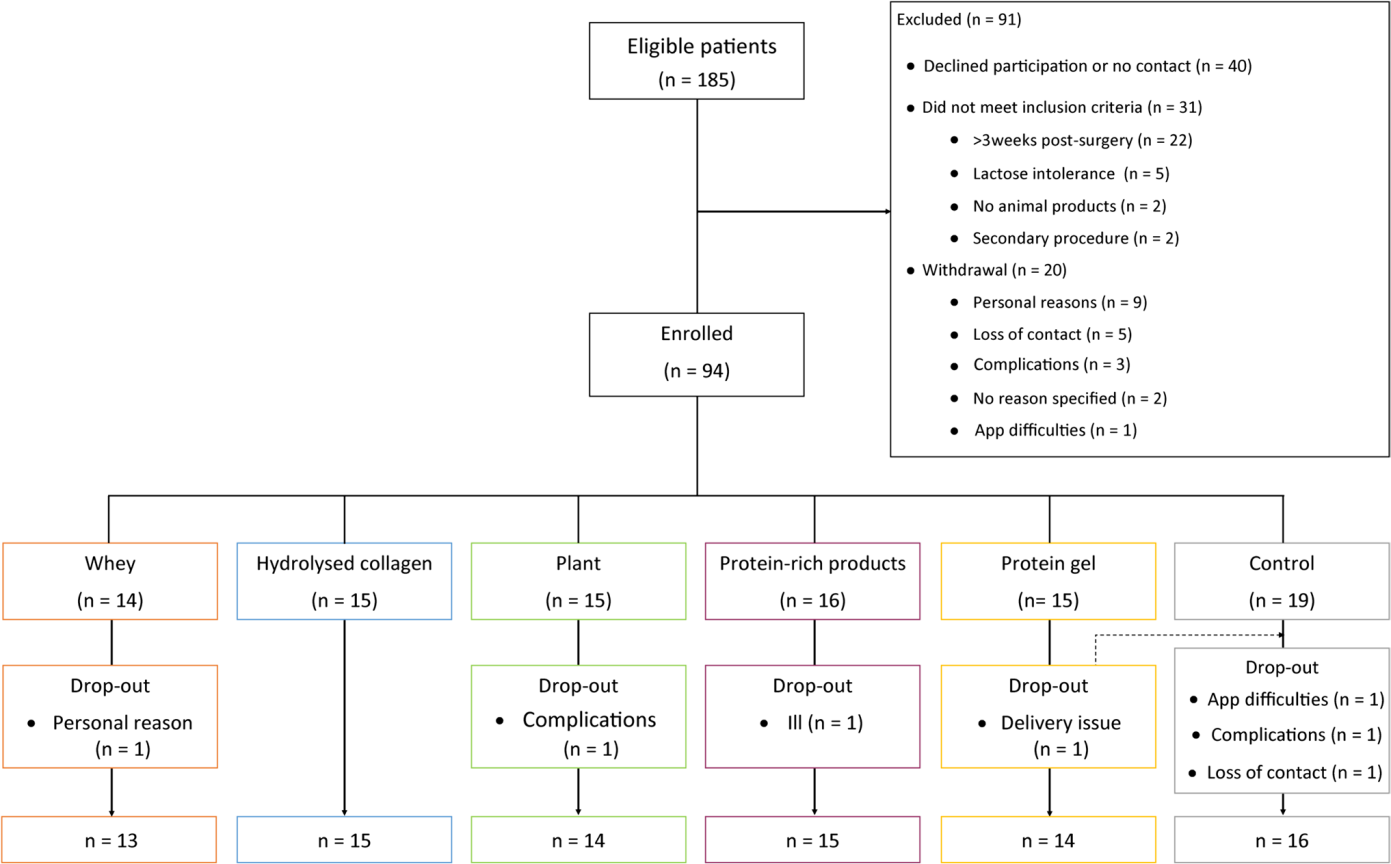
Participant flowchart: inclusion and drop-out

A total of 94 patients were enrolled, of which 7 dropped out or were lost to follow-up during the study (Fig. 2). The analytical cohort consisted of 87 participants, of whom dietary intake was available in 79 (91%) and 85 (98%) completed the questionnaire. Our population were mostly women (79%) aged 44 ± 12 years with a median BMI of 42.0 [39.6–47.5] kg/m^2^ (Table 2). Surgery type was comparable across study arms (*p* = 0.90). Baseline characteristics did not differ (Table 2).

**Table 2:** Baseline participant characteristics

Characteristic	PES					Control (*n* = 16)	*p*-value
	Whey (*n* = 13)	Hydrolysed collagen (*n* = 15)	Plant (*n* = 14)	Protein-rich products (*n* = 15)	Protein gel (*n* = 14)		
Age, years	45 ± 12	42 ± 12	43 ± 12	42 ± 13	42 ± 12	46 ± 12	0.90
18–30	2 (15%)	3 (20%)	3 (21%)	4 (27%)	3 (21%)	2 (13%)	
30–45	4 (31%)	6 (40%)	4 (29%)	3 (20%)	6 (43%)	4 (25%)	
45–60	6 (46%)	6 (40%)	7 (50%)	7 (47%)	5 (36%)	9 (56%)	
≥ 60	1 (8%)	0	0	1 (7%)	0	1 (6%)	
Sex, *n (%)*							
Female	10 (77%)	13 (87%)	12 (86%)	12 (80%)	11 (79%)	11 (69%)	0.85
Male	3 (23%)	2 (13%)	2 (14%)	3 (20%)	3 (21%)	5 (31%)	
Height, *m*	1.70 ± 0.13	1.73 ± 0.07	1.72 ± 0.08	1.74 ± 0.07	1.71 ± 0.09	1.72 ± 0.09	0.96
Weight, *kg*	125.9 [105.0–137.6]	126.5 [109.8–157.0]	124.2 [116.8–138.7]	129.3 [115.7–149.0]	125.3 [110.1–166.1]	119.3 [115.2–145.1]	0.94
BMI, *kg/m*^*2*^	41.8 [39.5–45.9]	42.0 [41.3–49.0]	41.9 [40.0–45.1]	43.8 [38.7–47.6]	40.9 [39.5–53.8]	42.0 [36.8–49.4]	0.99
<40	7 (39%)	3 (18%)	3 (21%)	5 (33%)	2 (20%)	3 (31%)	
40–50	9 (50%)	10 (59%)	10 (71%)	8 (53%)	4 (40%)	7 (54%)	
>50	2 (11%)	4 (24%)	1 (7%)	2 (13%)	4 (40%)	2 (15%)	
Surgery, *n (%)*							
Sleeve	8 (62%)	8 (53%)	8 (57%)	6 (40%)	8 (57%)	8 (50%)	0.90
Roux-en-Y gastric bypass	5 (38%)	7 (47%)	6 (43%)	9 (60%)	6 (43%)	8 (50%)	
Obesity-related health problems, *n (%)*							
None	8 (57%)	9 (64%)	8 (57%)	10 (67%)	8 (57%)	7 (44%)	0.85
Hypertension	6 (43%)	4 (29%)	3 (21%)	3 (20%)	4 (29%)	5 (31%)	
Dyslipidemia	1 (7%)	0	1 (7%)	2 (13%)	1 (7%)	3 (19%)	
Sleep apnea	3 (21%)	1 (7%)	2 (14%)	2 (13%)	2 (14%)	4 (25%)	
Arthrosis	1 (7%)	0	1 (7%)	1 (7%)	3 (21%)	1 (6%)	
Diabetes	1 (7%)	0	2 (14%)	1 (7%)	0	2 (13%)	
Dietary intake							
Energy, kcal/day	813 ± 269	722 ± 175	962 ± 346	917 ± 209	954 ± 364	880 ± 337	0.30
Protein, g/day	50.6 ± 21.9	46.9 ± 15.3	55.2 ± 21.2	57.5 ± 18.4	62.4 ± 29.5	56.3 ± 23.4	0.58

### Tolerability and User Satisfaction

**Fig. 3:**
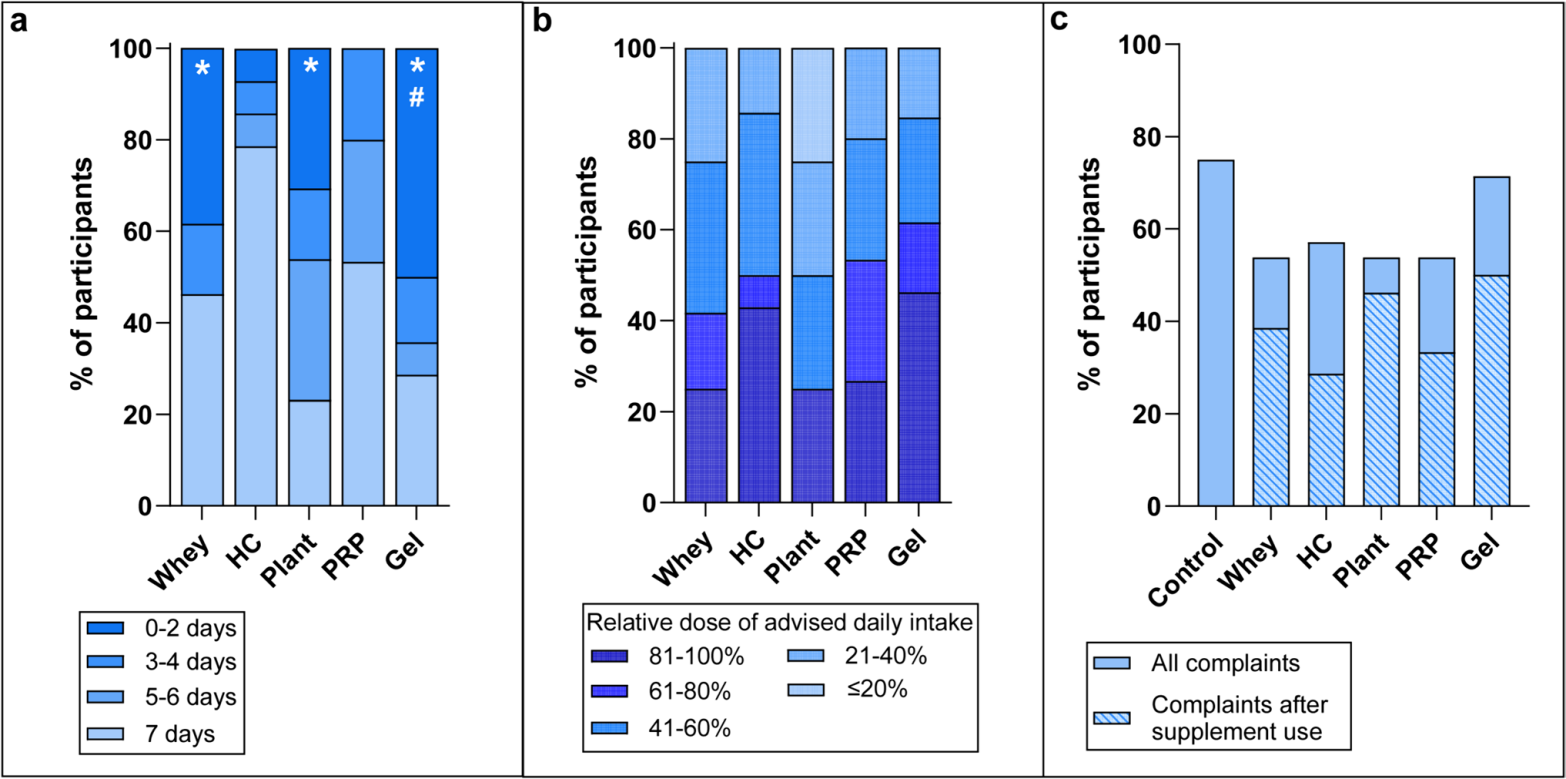
Tolerability and PES intake: Self-reported intake of protein-enhancing strategies with **a** number of days of protein-enhancing strategy use and **b** relative average daily intake of the protein-enhancing strategy to advised daily dosage and **c** self-reported dietary complaints after supplement use for the protein-enhancing strategies and overall dietary complaints not related to PES use for all study arms. HC, hydrolysed collagen; PRP, protein-rich products; Gel, protein gel. *Significant difference compared to HC; #significant difference compared to PRP

Intergroup and intragroup variability in PES use was found with 61% of participants reporting PES use of ≥ 5 days (range, 36 to 86%; Fig. 3a). Only 33% could consume > 80% of the advised daily intake of PES (Fig. 3b). Main reasons for PES disuse were dislike of taste (58%) and dietary complaints (24%), most commonly nausea. Most participants (61%) did not experience dietary complaints after PES use, and fewer PES participants reported dietary complaints than controls (Fig. 3c).

**Fig. 4:**
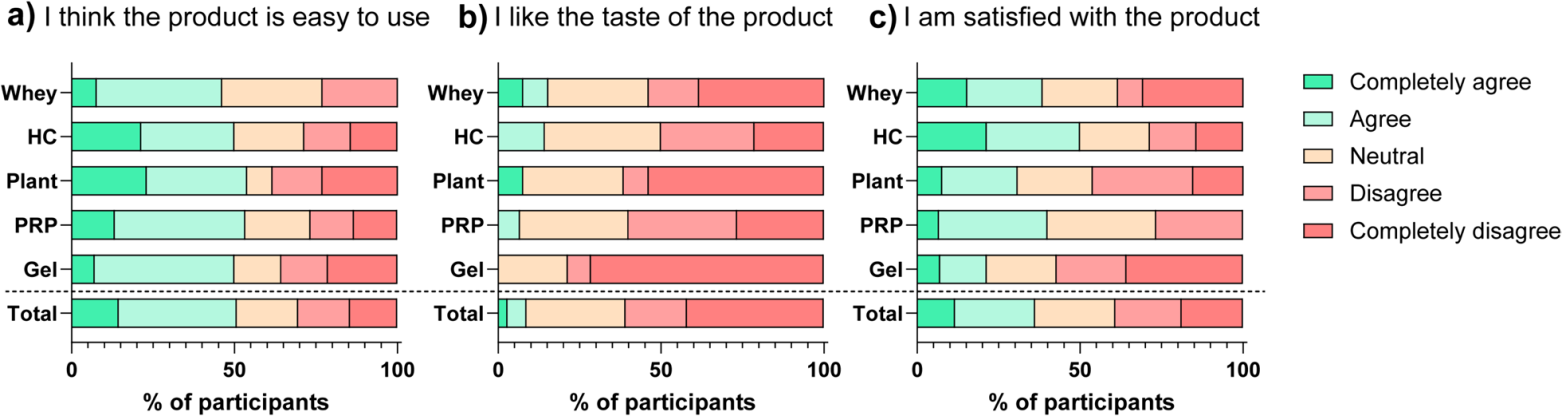
User satisfaction with protein-enhancing strategies for three statements on a 5-point Likert scale: ease of use, taste and overall satisfaction. HC, hydrolysed collagen; PRP, protein-rich products; Gel, protein gel

User satisfaction was also variable (Fig. 4, Supplement 5). According to 70% of participants, PES were easy to use, but 61% disliked the taste. Overall satisfaction was highest for HC, PRP and whey (71%, 73% and 62%, respectively; Fig. 4c).

### Dietary Protein Intake

**Fig. 5:**
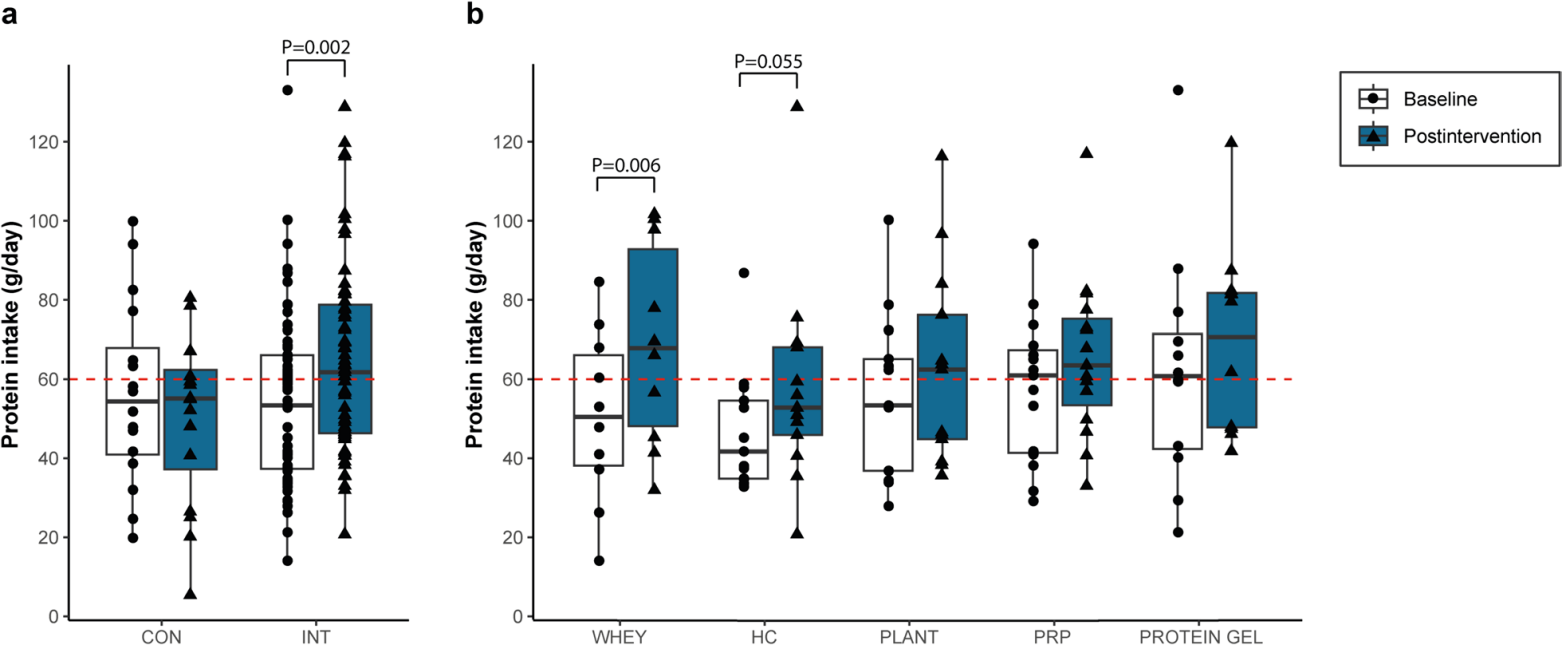
Overall daily protein intake at baseline and intervention week for **a** the protein-enhancing strategies combined and control and **b** subdivided per protein-enhancing strategy. The red line depicts the advised protein intake of 60 g per day according to current guidelines. Bar charts showing median and interquartile range. CON, control; INT, PES arms combined; HC, hydrolysed collagen; PRP, protein-rich products

At baseline, participants consumed 877 ± 294 kcal/day with 55.0 ± 21.7 g of protein per day. Baseline energy and protein intake were similar for control and PES (Supplement 6). No significant changes in energy or protein intake were observed in the control group between baseline and intervention (880 ± 337 to 800 ± 379 kcal/day, *p* = 0.50 and 56.3 ± 23.4 to 50.0 ± 21.3 g of protein per day, *p* = 0.33). PES combined significantly increased protein intake from baseline to intervention (54.7 ± 21.5 to 64.7 ± 23.4 g/day, *p* = 0.002), while energy intake remained unchanged (876 ± 286 to 896 ± 302 kcal/day, *p* = 0.86). Protein intake was significantly higher for PES compared to control during the intervention (*p* = 0.025; Fig. 5a, Supplement 7). PES groups were more likely to reach a protein intake ≥ 60 g in the intervention week compared to control (52% vs. 31% of participants, odds ratio 4.2 [0.8–21.4]). For PES subgroups, whey significantly increased their protein intake from baseline to intervention, and a trend was found for HC (Fig. 5b, Supplement 8). The absolute protein intake during the intervention was significantly higher for whey, PRP and protein gel compared to control (*p* = 0.052, *p* = 0.048 and *p* = 0.026, respectively; Supplement 7). Similar outcomes were observed for ∆protein intake before and after correction for baseline protein intake (Supplement 8).

## Discussion

Supplements are considered a solution for insufficient protein intake post-MBS, but tolerability of these supplements is insufficiently studied. The aim of this study was to evaluate tolerability and satisfaction with five different PES and their effect on protein intake shortly after MBS. Our results show that participants experienced less dietary complaints after consuming PES than from their regular diet and, the majority were able to use PES ≥ 5 days during the intervention. However, we found dissatisfaction with PES taste which was also the main reason to discontinue PES use. Still, on average, an increase in protein intake with PES was found as participants in the intervention arms were more likely to reach the ≥ 60 g daily protein intake advised by guidelines [5]. These findings suggest that PES can improve protein intake shortly post-MBS.

Tolerability of PES was defined as the ability to consume PES in the advised dosage without experiencing more dietary complaints than with the habitual diet. PES caused less dietary complaints in our study than the habitual diet. As much as 61% of the participants could use PES ≥ 5 days but only one-third could consume > 80% of the advised daily intake. Other studies in post-MBS populations also found low compliance to protein supplements but did not explore its causes [31, 33]. One study reported on tolerability of ready-to-drink protein: 45% of the participants could use > 80% of the supplemental drink (2 drinks of 30 g protein) and 73% could consume 1 drink at 4–12 weeks post-bariatric surgery with 40% of the participants reporting dislike of taste [16]. In the present study, a higher dislike of taste (61% of participants) for all PES was found, while 70% of participants found PES easy to use. Palatability may play a role in limiting PES intake post-MBS, so improving taste and therefore satisfaction could benefit tolerability of PES.

Changes in protein digestion and taste that occur in patients after MBS combined with the restrictive nature of MBS challenge the consumption of sufficient protein. Literature shows variable protein intakes with protein supplements shortly post-bariatric surgery from 78.3 ± 27.9 g/day at 3 weeks post-surgery to 36.0 ± 15.4 g/day at 1 month post-surgery [16, 33]. Differences between studies may be caused by cultural or care differences [16, 33]. We found that PES can increase daily protein intake in 70% of the PES users to an average protein intake of 64.7 ± 23.4 g per day, while only 38% of the control group increased their protein intake. Protein intakes above recommendation were reached by 54% of the PES users compared to 31% for control [5]. Further research needs to explore ways to increase protein intake above guideline for all patients shortly post-MBS as sufficient protein intake has been shown to decrease lean tissue mass loss relative to total weight loss [24].

When comparing PES, we find differences in tolerability, satisfaction and protein intake. HC performs best on tolerability and satisfaction which was expected as hydrolysed protein is thought to be more easily digested post-bariatric surgery [7]. In the HC arm, 86% of the participants could use HC ≥ 5 days, 43% reached > 80% of the advised daily intake and 71% was satisfied with the product. Only protein gel did better regarding advised daily intake as > 80% was reached by 46%. Yet, whey results in the highest increase in protein intake and significant differences compared to control. PES use may create further awareness of daily protein intake and increase the intake of habitual dietary products with a high protein concentration as in our study PES were most often mixed with dairy products.

Not one PES stands out in tolerability, satisfaction and effect on protein intake. Taste is an issue for all PES regardless of protein source or product type. Nevertheless, the results of this study lean towards a preference for the use of HC shortly after MBS to increase protein intake. HC is tolerated best which was expected due to its hydrolysed nature. HC also shows a near-significant increase in protein intake despite not reaching a daily protein intake of 60 g per day. However, current research shows no benefits of collagen supplementation on muscle protein synthesis or the loss of lean mass [3, 17, 29], but collagen could benefit other post-MBS issues like bone health and wound healing [6, 8]. Therefore, HC may need to be combined with another protein source that stimulates muscle protein synthesis to prevent FFM loss. The other PES also show promise for improving protein intake shortly post-MBS if improvements in taste and therefore tolerability could be made. Furthermore, when taking into account the high interindividual variability in recovery after MBS, the role of surgery type, and personal preferences regarding diet, PES may not be a one-size-fits-all strategy and needs to be further researched.

### Strengths and Limitations

This study is the first to assess tolerability of PES shortly post-MBS. We adopted a real-world study design which permits realistic assessment of patient behaviour that is applicable to clinical practice. Our study was not randomised due to practical considerations, and more patients with sleeve gastrectomy than Roux-en-Y gastric bypass were included (53% vs. 47%) compared to Dutch reference values (21% vs. 53%) [11]. Nevertheless, baseline characteristics were similar across study arms (Table 2). To also limit the effect of surgery type, we scheduled the study in weeks 3 and 4 post-MBS as both surgery types will be able to consume a solid diet [20]. Dietary data and questionnaires were self-reported so may be subject to recall and reporting bias. Nevertheless, we used a validated mobile application to collect dietary intake data which limits the magnitude of bias [20]. Also, only participants who reported dietary intake for ≥ 2 days each week with at least three completed dietary logs per day were included in our study.

## Conclusion

Overall, an improved protein intake with PES use was seen. However, not one PES in particular stands out in both its effect on tolerability, satisfaction and protein intake. HC seems to be the most tolerable PES, while whey shows the largest increase in protein intake. Taste seems to be the biggest challenge regarding PES use in the population post-bariatric surgery.

## Supplementary Information

Below is the link to the electronic supplementary material.

Supplementary file1 (PNG 318 KB)

Supplementary file2 (DOCX 33 KB)

Supplementary file3 (PNG 135 KB)

Supplementary file4 (PNG 195 KB)

Supplementary file5 (PNG 233 KB)

Supplementary file6 (PNG 251 KB)

## Data Availability

The data that support the findings of this study are available from the corresponding author upon reasonable request.
