# Supplementary figures and images for: Protein Supplement Tolerability and Patient Satisfaction after Bariatric Surgery
Source: Obes Surg. 2024 Sep 7;34(10):3866–75. doi: 10.1007/s11695-024-07462-4 (PMC11481670; doi:10.1007/s11695-024-07462-4)

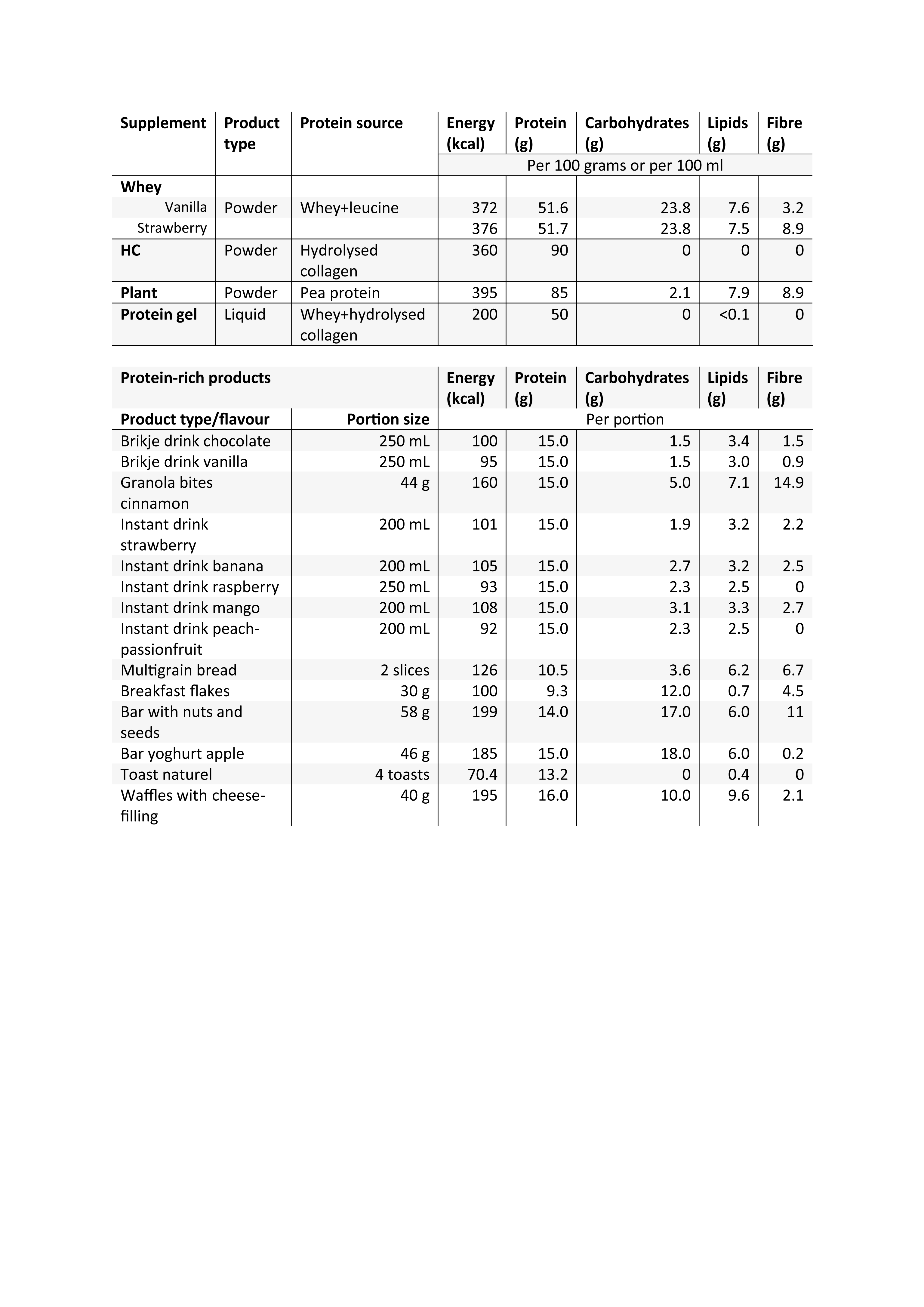

Supplement: Supplementary file 1 — Supplementary file1 (PNG 318 KB) [file 11695_2024_7462_MOESM1_ESM.png]

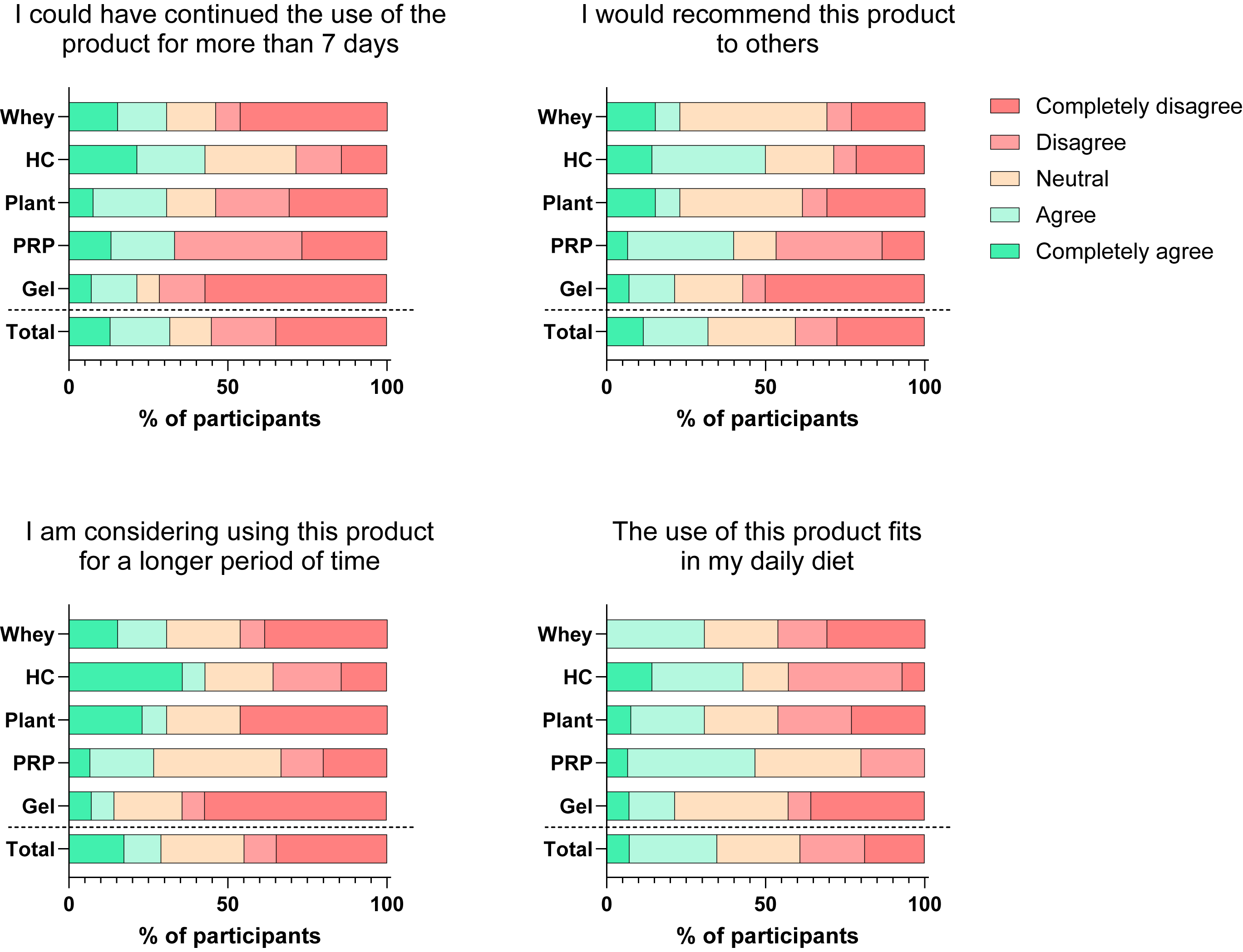

Supplement: Supplementary file 3 — Supplementary file3 (PNG 135 KB) [file 11695_2024_7462_MOESM3_ESM.png]

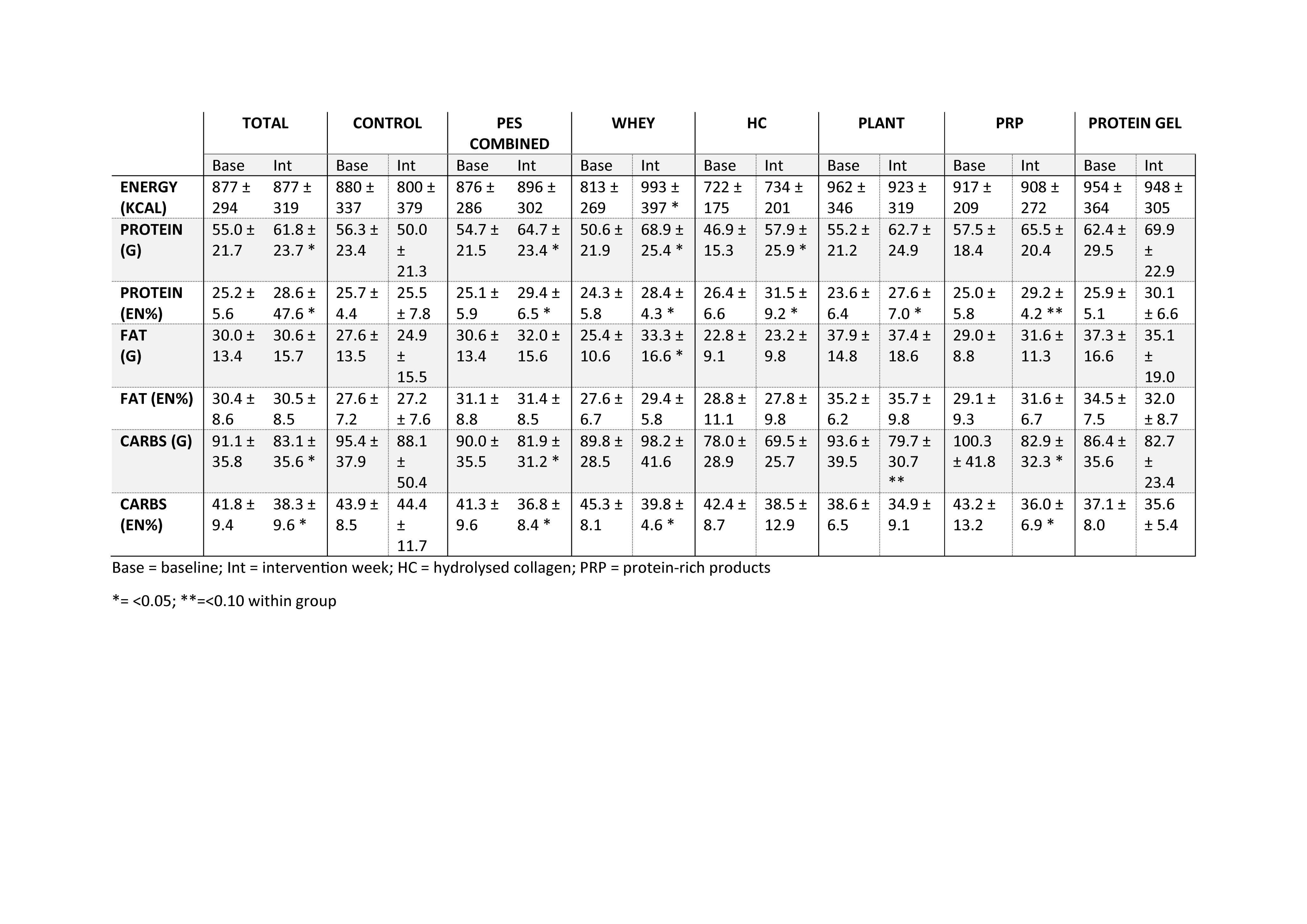

Supplement: Supplementary file 4 — Supplementary file4 (PNG 195 KB) [file 11695_2024_7462_MOESM4_ESM.png]

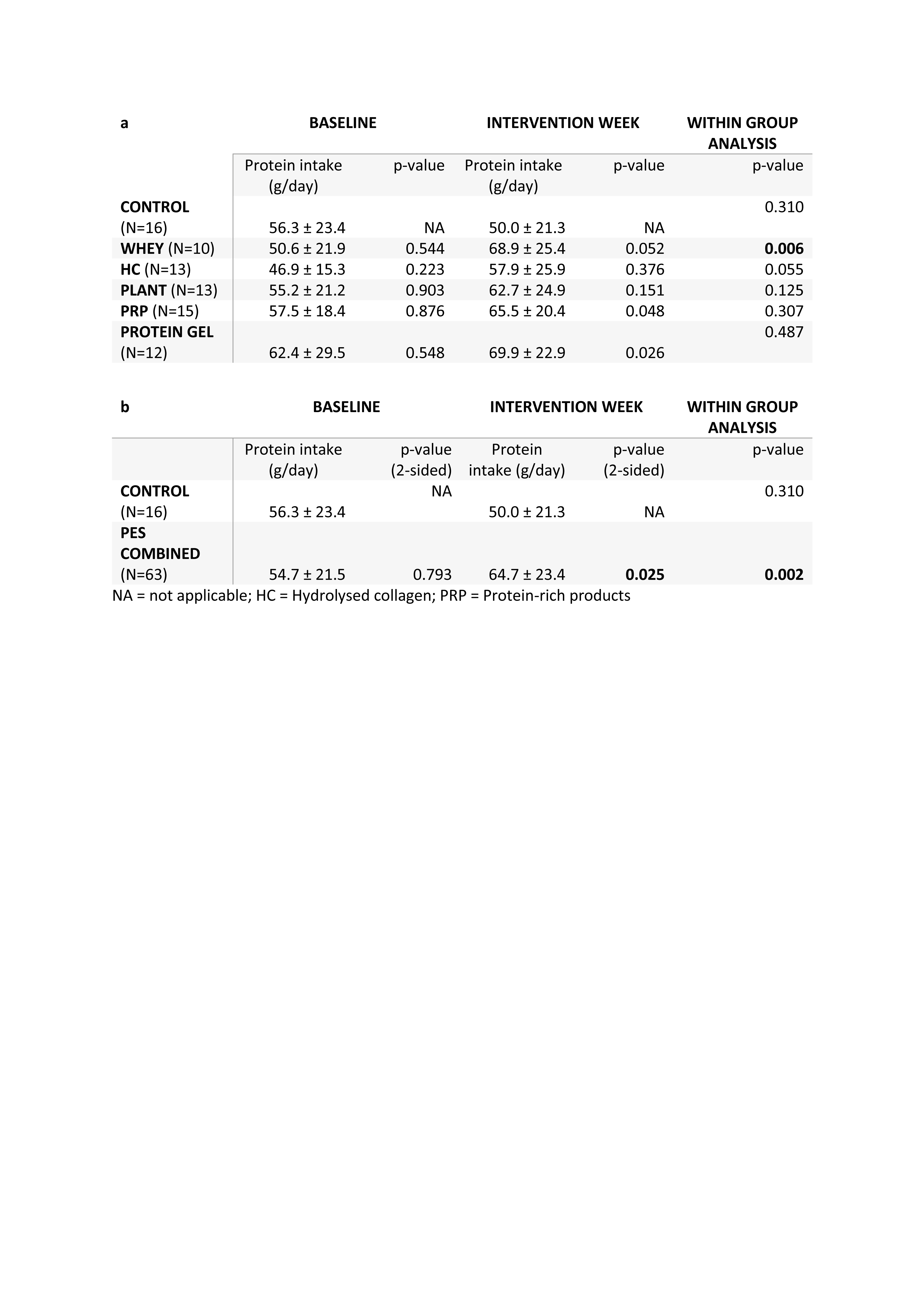

Supplement: Supplementary file 5 — Supplementary file5 (PNG 233 KB) [file 11695_2024_7462_MOESM5_ESM.png]

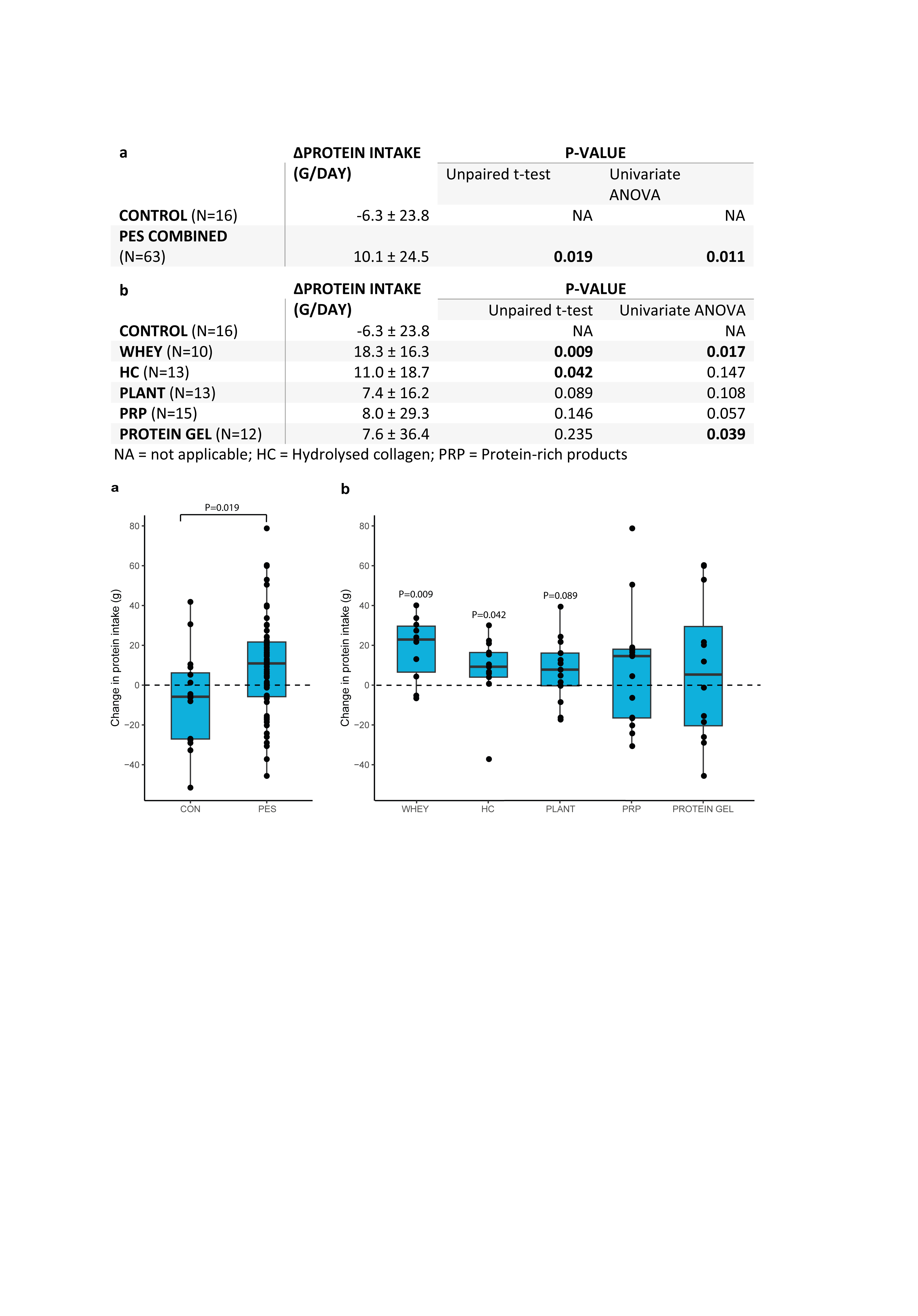

Supplement: Supplementary file 6 — Supplementary file6 (PNG 251 KB) [file 11695_2024_7462_MOESM6_ESM.png]
