# Supplementary material for: Protein Supplement Tolerability and Patient Satisfaction after Bariatric Surgery
Source: Obes Surg. 2024 Sep 7;34(10):3866–75. doi: 10.1007/s11695-024-07462-4 (PMC11481670; doi:10.1007/s11695-024-07462-4)
Supplement: Supplementary file 2 — Supplementary file2 (DOCX 33 KB) [file 11695_2024_7462_MOESM2_ESM.docx]

**Supplements**

Supplemental table 1: Nutritional information of the five protein supplements

Supplement 2-4: see page 2 through 18.

Supplement 5: Four statements regarding satisfaction with PES on a 5-Point Likert scale

Supplemental table 6: Macronutrient intake at baseline and intervention week per group

Supplemental table 7 - Protein intake of protein-enhancing strategies compared to control with an unpaired t-test and within group analysis with paired t-test

Supplement 8 - Analysis of change in protein intake between intervention week and baseline from protein-enhancing strategies compared to control. Univariate ANOVA with baseline protein intake as confounder.

Supplemental figure 8 – Change in protein intake from baseline to intervention with (a) showing the change for control and the protein-enhancing strategies combined and (b) subdivided per protein-enhancing strategy with p-value showing t-test p-value with control as reference group. Bar-charts showing median and interquartile range. CON = control; INT = intervention; HC = hydrolysed collagen; PRP = protein-rich products.

Supplement 2 - Short questionnaire during dietary intake logs

1. Heeft u de afgelopen uren klachten gehad na voedselinname? / Did you experience dietary complaints the last couple of hours?

o Ja / Yes

o Nee / No

Display This Question If 1. = Ja / Yes

2. Welke klachten heeft u de afgelopen uren gehad? / Which dietary complaints did you experience the last couple of hours?

▢ Misselijkheid / Nausea

▢ Braken / Vomiting

▢ Zuurbranden / Heartburn

▢ Buikpijn / Stomachache

▢ Vol gevoel / Full feeling

▢ Darmklachten / Intestinal complaints

▢ Verandering in ontlasting / Change in stool

▢ Anders / Other

Display This Question If 2. = Anders / Other

3. Anders, namelijk / Other, namely

________________________________________________________________

Display This Question If 1. = Ja / Yes

4. Hoe lang duurden de klachten? / How long did the dietary complaints last?

o <15 minuten / <15 minutes

o 15 tot 30 minuten / 15 to 30 minutes

o 30 tot 60 minuten / 30 to 60 minutes

o >60 minuten / >60 minutes

Display This Question If 1. = Ja / Yes

5. Kunt u de klachten verklaren door de manier van eten (te snel, teveel, te grote happen/slokken)? / Can the dietary complaints be explained by the way of eating (too fast, too much, too large bites/sips)?

o Ja / Yes

o Nee / No

6. Heeft u de afgelopen uren het eiwitproduct ingenomen? / Did you use the protein products the last couple of hours?

o Ja / Yes

o Nee / No

Supplement 3 - Final questionnaire control group

1. Heeft u de afgelopen week last gehad van onderstaande klachten na voedselinname? / Did you experience dietary complaints after food intake during the last week?
   1. Ik heb geen klachten gehad / I did not experience complaints
   2. Misselijkheid / Nausea
   3. Braken / Vomiting
   4. Zuurbranden / Heartburn
   5. Buikpijn / Stomachache
   6. Vol gevoel / Full feeling
   7. Darmklachten / Intestinal complaints
   8. Verandering in ontlasting / Change in stool
   9. Dumping (zweten, hartkloppingen, vermoeidheid) / Dumping (sweating, palpitations, fatigue)
   10. Vastloper / Dysphagia
   11. Ik had andere klachten / I experienced other complaints

If 1. = “Ik had andere klachten” / “I experienced other complaints”

1. Welke andere klachten had u? / Which complaints did you experience?

_____________________________

If 1. ≠ “Ik heb geen klachten gehad” / “I did not experience complaints”

1. Van welke producten kreeg u deze klachten? / Which dietary products gave you these complaints?

_____________________________

If 1. ≠ “Ik heb geen klachten gehad” / “I did not experience complaints”

1. Kunt u de klachten na voedselinname verklaren door de manier van eten (te snel, teveel, te grote happen/slokken etc.)? / Can you explain the complaints by the way of consuming the product (too fast, too much, bites too large etc.)?
   1. Ja / Yes
   2. Nee / No

If 1. ≠ “Ik heb geen klachten gehad” / “I did not experience complaints”

1. Hoe lang duurden de klachten na inname gemiddeld? / How long did the complaints last?
   1. <15 minuten / <15 minutes
   2. 15-30 minuten / 15-30 minutes
   3. 30-60 minuten / 30-60 minutes
   4. >60 minuten / >60 minutes

If 1. ≠ “Ik heb geen klachten gehad” / “I did not experience complaints”

1. Zijn de klachten na voedselinname gedurende de week veranderd? / Did the dietary complaints after food intake change during the week?

-5______0______5

If 1. ≠ “Ik heb geen klachten gehad” / “I did not experience complaints”

1. Zijn de klachten die u had voor u een reden om het product niet meer te gebruiken? / Were the dietary complaints for you reason to stop using the product that gave the complaints?
   1. Ja / Yes
   2. Nee / No

If 7. = “Ja” / “Yes”

1. Kunt u uw antwoord toelichten? / Can you explain your answer?

___________________________

1. Mogen wij u benaderen met aanvullende vragen over het onderzoek of het gebruik van de app? / Can we contact you with additional research questions or questions about the use of the mobile application?
   1. Ja / Yes
   2. Nee / No
2. Mogen wij u in de toekomst benaderen om deel te nemen aan een kort onderzoek naar het gebruik van een beweegapp? / Can we contact you in the future to participate in a short study researching the use of a fitness app?
   1. Ja / Yes
   2. Nee / No

Supplement 4 - Final questionnaire – Whey, Plant, Hydrolysed collagen, Protein gel

**Inname / Intake**

1. Op welke datum bent u begonnen met de inname van het eiwitproduct? / On which date did you start using the protein product?

dd-m-yyyy

1. Hoeveel dagen van de week heeft u het eiwitproduct (of een deel hiervan) ingenomen? / How many days of the week did you use the protein product or a part of the protein product?
   1. 0 dagen / 0 days
   2. 1 dag / 1 day
   3. 2 dagen / 2 days
   4. 3 dagen / 3 days
   5. 4 dagen / 4 days
   6. 5 dagen / 5 days
   7. 6 dagen / 6 days
   8. 7 dagen / 7 days

If 2. ≠ “7 dagen” / “7 days”

1. Waarom is het op sommige dagen niet gelukt om het eiwitproduct in te nemen? / Why were you unable to use the protein product on some days?
   1. Ik kreeg klachten door het supplement (bijv. misselijkheid, dumping) / I got complaints from the supplement (e.g. nausua, dumping)
   2. Ik vond het supplement niet lekker / I didn’t like the taste of the supplement
   3. Ik had een vol gevoel / I had a full feeling
   4. Ik was vergeten om het supplement in te nemen / I forgot to take the supplement
   5. Ik had last van smaak-geurverandering na de operatie / I experienced changes in taste and smell after surgery
   6. Ik had geen eetlust/geen dorst / I wasn’t hungry or thirsty
   7. Ik vond het spannend om (het product) te eten / I was tensive to using the product
   8. Anders / Other

If 3. = “Anders” / “Other”

1. Anders, namelijk / Other, namely

___________________

If 2. ≠ “0 dagen” / “0 days”

1. Hoeveel van de bijgeleverde (scoop)lepels van het eiwitproduct heeft u dagelijks in totaal op? / “How many of the supplied scoops did you use daily of the protein product?
   1. 1 lepel (+-5 gram) / 1 spoon
   2. 2 lepels (+-10 gram) / 2 spoons
   3. 3 lepels (+-15 gram) / 3 spoons
   4. 4 lepels (+-20 gram) / 4 spoons
   5. 5 lepels (+-25 gram) / 5 spoons
   6. 6 lepels (+-30 gram) / 6 spoons

- Antwoordopties voor protein gel: / Answeroptions for the protein gel
  - Half zakje (10 gram) / Half a satchet
  - 1 zakje (20 gram) / 1 satchet
  - 1,5 zakje (25 gram) / 1.5 satchet
  - 2 zakjes / 2 satchets

If 2. ≠ “0 dagen” / “0 days”

1. In hoeveel porties per dag heeft u het eiwitproduct ingenomen? / In how many daily portions did you take in the protein product?
   1. 1 portie / 1 portion
   2. 2 porties / 2 portions
   3. 3 porties / 3 portions
   4. Meer dan 3 porties / more than 3 portions

If 2. ≠ “0 dagen” / “0 days”

1. Portiegrootte: In welke stelling herkent u zich het meest? / Portionsize: which statement do you agree with most?
   1. Ik had elk innamemoment een gelijke portie / I used a similar portion at each moment I used the protein product
   2. Ik nam de grootste portie in de ochtend / I took the biggest portion in the morning
   3. Ik nam de grootste portie in de middag / I took the biggest portion in the afternoon
   4. Ik nam de grootste portie in de avond / I took the biggest portion in the evening

If 2. ≠ “0 dagen” / “0 days”

1. Hoe lang deed u gemideld over de inname van één portie? / How long did it take to take in one portion?

____ minuten / ____ minutes

If 2. ≠ “0 dagen” / “0 days”

1. Hoe heeft u het eiwitproduct gebruikt? / How did you use the protein product?
   1. Als aanvulling op de maaltijd / In addition to a meal
   2. Als maaltijvervanger / As meal replacement
   3. Als tussendoortje / As a snack

If 2. ≠ “0 dagen” / “0 days”

1. Op welke manier heeft u het eiwitproduct ingenomen? / How did you take in the protein product?
   1. Opgelost in water / Dissolved in water
   2. Opgelost in ranja / Dissolved in lemonade
   3. Opgelost in een zuivelproduct (bijv. melk, drinkyoghurt, kwark, Griekse yoghurt) / Dissolved in a dairy product (e.g. milk, drinkyoghurt, soft cheese, Greek yoghurt)
   4. Opgelost in koffie / Dissolved in coffee
   5. Anders / Other

If 10. = “Anders” / “Other”

1. Anders, namelijk / Other, namely

_________________

**Tevredenheid / Satisfaction**

Antwoordopties voor onderstaande stellingen op basis van 5-Point Likert scale: helemaal mee oneens, mee oneens, neutraal, mee eens, helemaal mee eens / Answeroptions for the statements below on a 5-Point Likert scale: completely disagree, disagree, neutral, agree, completely agree

1. Ik vind het eiwitproduct makkelijk te gebruiken. / I thought the protein products was easy to use
2. Ik vind de smaak van het eiwitproduct lekker. / I like the taste of the protein product
3. Ik had de inname van het eiwitproduct langer vol kunnen houden dan 7 dagen. / I could have used the protein product for a longer period than 7 days
4. Ik vind de inname van het eiwitproduct passen binnen mijn dagelijkse voedingspatroon. / I think the use of the protein product fits in my daily diet
5. Ik overweeg na dit onderzoek door te gaan met het innemen van het eiwitproduct. / I consider using the protein product after the research period has ended
6. Over het algemeen ben ik tevreden met het gebruik van dit eiwitproduct. / I am satisfied with the use of the protein product
7. Ik zou dit eiwitproduct aanraden aan een ander. / I woudl recommend this protein product to others

**Klachten / Complaints**

1. Heeft u de afgelopen week na inname van het eiwitproduct last gehad van onderstaande klachten? / Did you experience dietary complaints after using the protein product during the last week?
   1. Ik heb geen klachten gehad / I did not experience complaints
   2. Misselijkheid / Nausea
   3. Braken / Vomiting
   4. Zuurbranden / Heartburn
   5. Buikpijn / Stomachache
   6. Vol gevoel / Full feeling
   7. Darmklachten / Intestinal complaints
   8. Verandering in ontlasting / Change in stool
   9. Dumping (zweten, hartkloppingen, vermoeidheid) / Dumping (sweating, palpitations, fatigue)
   10. Vastloper / Dysphagia
   11. Ik had andere klachten / I experienced other complaints

If 1. = “Ik had andere klachten” / “I experienced other complaints”

1. Welke andere klachten had u? / Which complaints did you experience?

_____________________________

If 1. ≠ Ik heb geen klachten gehad / “I did not experience complaints”

1. Hoe lang duurden de klachten na iname van het eiwitproduct gemiddeld? / How long did the complaints after using the protein product last on average?
   1. <15 minuten / <15 minutes
   2. 15-30 minuten / 15-30 minutes
   3. 30-60 minuten / 30-60 minutes
   4. >60 minuten / >60 minutes

If 1. ≠ Ik heb geen klachten gehad / “I did not experience complaints”

1. Kunt u de klachten na inname van het eiwitproduct verklaren door de manier van eten (te snel, teveel, te grote happen/slokken etc.)? / Can you explain the complaints after consumption of the protein product by the way of eating (too fast, too much, too large bites etc.)?
   1. Ja / Yes
   2. Nee / No

If 1. ≠ Ik heb geen klachten gehad / “I did not experience complaints”

1. Zijn de klachten van het eiwitproduct gedurende de week veranderd? / Did the complaints from the protein product change during the week?
   1. De klachten zijn gelijk gebleven / The complaints did not change
   2. De klachten zijn verminderd / The complaints diminished
   3. De klachten zijn verergerd / The complaints worsened

If 1. ≠ Ik heb geen klachten gehad / “I did not experience complaints”

1. In hoeverre denkt u dat de klachten die u had veroorzaakt worden door het eiwitproduct? / Do you think the protein product caused the complaints?
   1. Zeer onwaarschijnlijk / Highly unlikely
   2. Onwaarschijnlijk / Unlikely
   3. Niet waarschijnlijk / niet onwaarschijnlijk // Not likely / not unlikely
   4. Waarschijnlijk / Likely
   5. Zeer waarschijnlijk / Highly likely

If 1. ≠ Ik heb geen klachten gehad / “I did not experience complaints”

1. Zijn de klachten die u had voor u een reden om het eiwitproduct niet meer te gebruiken? / Were the complaints reason to stop using the protein product?
   1. Ja / Yes
   2. Nee / No
2. Zijn er andere producten in uw voeding waar u regelmatig klachten van krijgt? / Are there other products in your diet that regularly cause complaints?
   1. Ja / Yes
   2. Nee / No

If 8. = Ja / “Yes”

1. Welke producten zijn dit? En welke klachten krijgt u? Which products? And which complaints do you get?

_______________________________

If 8. = Ja / “Yes”

1. Zijn de klachten na inname van deze voedingsmiddelen gedurende de week veranderd? / Did the complaints from those products change during the week?
   1. Ik heb deze voedingsmiddelen deze week niet ingenomen / I did not use those products this week
   2. De klachten zijn gelijk gebleven / The complaints did not change
   3. De klachten zijn verminderd / The complaints diminished
   4. De klachten zijn verergerd / The complaints worsened
2. Heeft u naar aanleiding van het onderzoek of de vragenlijst opmerkingen of vragen? / Do you have any remarks or questions regarding the study or questionnaire?

_________________________________

1. Mogen wij u benaderen met aanvullende vragen over het onderzoek of het gebruik van de app? / Can we contact you with questions about the study or the use of the mobile application?
   1. Ja / Yes
   2. Nee / No
2. Mogen wij u in de toekomst benaderen om deel te nemen aan een kort onderzoek naar het gebruik van een beweegapp? / Can we contact you in the future to ask you to take part in a study looking into the use of a fitness app
   1. Ja / Yes
   2. Nee / No

Final questionnaire – Protein-rich products

**Inname**

1. Op welke datum bent u begonnen met de inname van de eiwitproducten? / On which date did you start using the protein product?

dd-m-yyyy

1. Hoeveel dagen van de week heeft u de eiwitproducten (of een deel hiervan) ingenomen? / How many days of the week did you use the protein product or a part of the protein product?
   1. 0 dagen / 0 days
   2. 1 dag / 1 day
   3. 2 dagen / 2 days
   4. 3 dagen / 3 days
   5. 4 dagen / 4 days
   6. 5 dagen / 5 days
   7. 6 dagen / 6 days
   8. 7 dagen / 7 days

If 2. ≠ “7 dagen” / “7 days”

1. Waarom is het op sommige dagen niet gelukt om de eiwitproducten in te nemen? / Why were you unable to use the protein product on some days?
   1. Ik kreeg klachten door het supplement (bijv. misselijkheid, dumping) / I got complaints from the supplement (e.g. nausua, dumping)
   2. Ik vond het supplement niet lekker / I didn’t like the taste of the supplement
   3. Ik had een vol gevoel / I had a full feeling
   4. Ik was vergeten om het supplement in te nemen / I forgot to take the supplement
   5. Ik had last van smaak-geurverandering na de operatie / I experienced changes in taste and smell after surgery
   6. Ik had geen eetlust/geen dorst / I wasn’t hungry or thirsty
   7. Ik vond het spannend om (het product) te eten / I was tensive to using the product
   8. Anders / Other

If 3. = “Anders” / “Other”

1. Anders, namelijk / Other, namely

_____________________

If 2. ≠ “0 dagen” / “0 days”

1. Welke producten heeft u deze week ingenomen? / Which products did you use this week?
   1. Brikje drank chocolade / Brikje drink chocolate
   2. Brikje drank vanille / Brikje drink vanilla
   3. Granola bites kaneel / Granola bites cinnamon
   4. Instant drink aardbei / Instant drink strawberry
   5. Instant drink banaan / Instant drink banana
   6. Instant drink framboos / Instant drink raspberry
   7. Instant drink mango / Instant drink mango
   8. Instant drink perzik-passievrucht / Instant drink peach-passionfruit
   9. Multigranenbrood / Multigrain bread
   10. Ontbijtflakes / Breakfast cereal
   11. Reep met noten en zaden / Bar with nuts and seeds
   12. Reep yoghurt appel / Bar yogurt apple
   13. Toast natuur / Toast
   14. Wafeltjes met kaasvulling / Wafers with cheesefilling

If 2. ≠ “0 dagen” / “0 days”

1. Op welk moment van de dag nam u de eiwitproducten? / On which moment of the day did you use the protein product?
   1. In de ochtend / In the morning
   2. In de middag / In the afternoon
   3. In de avond / In the evening

If 2. ≠ “0 dagen” / “0 days”

1. Hoe gebruikte u de eiwitproducten voornamelijk? / How did you use the protein product?
   1. Als aanvulling op de maaltijd / In addition to a meal
   2. Als maaltijvervanger / As meal replacement
   3. Als tussendoortje / As a snack

If 2. ≠ “0 dagen” / “0 days”

1. Indien u 1 van de repen heeft gegeten: heeft u de reep in zijn gehele vorm kunnen eten? / If you consumed one of the bars: were you able to eat the entire bar?
   1. Ja, ik heb de gehele reep in 1 eetmoment gegeten / Yes, I ate the bar in one meal
   2. Nee, ik heb de reep in stukken gesneden en verdeeld over meerdere eetmomenten / No, I cut the bar in parts en divided them over multiple meals
   3. Ik heb geen reep op / I did not eat a bar

If 8. = “Nee, ik heb de reep in stukken gesneden en verdeeld over meerder eetmomenten” / “No, I cut the bar in parts en divided them over multiple meals”

1. Over hoeveel eetmomenten heeft u de reep verspreid? / Over how many meals did you spread the bar?

__________

If 2. ≠ “0 dagen” / “0 days”

1. Indien u 1 van de drankjes heeft geconsumeerd: heeft u de drank in zijn geheel in 1 drinkmoment kunnen innemen? / If you used one of the drinks: were you able to drink it whole?
   1. Ja / Yes
   2. Nee / No
   3. Ik heb geen drankje op / I did not use a drink

If 10. = “Nee” / “No”

1. Over hoeveel drinkmomenten heeft u de drank moeten verspreiden? / Over how many moments did you spread the drink?

____________

If 2. ≠ “0 dagen” / “0 days”

1. Indien u de granola of ontbijtflakes heeft geconsumeerd: heeft u de ontbijtgranen los gegeten of heeft u deze gecombineerd met een ander product (zoals melk of yoghurt)? / If you used the granola or cereal: did you eat them by itself or combined with another product (e.g. milk or yogurt)?
   1. Los kunnen eten / Ate them by itself
   2. Gecombineerd met melk/yoghurt / Combined with milk/yogurt
   3. Beide / Both
   4. Ik heb geen granola of ontbijtflakes op / I did not use the granola or cereal

**Tevredenheid**

Antwoordopties voor onderstaande stellingen op basis van 5-Point Likert scale: helemaal mee oneens, mee oneens, neutraal, mee eens, helemaal mee eens / Answeroptions for the statements below on a 5-Point Likert scale: completely disagree, disagree, neutral, agree, completely agree

1. Ik vind het eiwitproduct makkelijk te gebruiken. / I thought the protein products was easy to use
2. Ik vind de smaak van het eiwitproduct lekker. / I like the taste of the protein product
3. Ik had de inname van het eiwitproduct langer vol kunnen houden dan 7 dagen. / I could have used the protein product for a longer period than 7 days
4. Ik vind de inname van het eiwitproduct passen binnen mijn dagelijkse voedingspatroon. / I think the use of the protein product fits in my daily diet
5. Ik overweeg na dit onderzoek door te gaan met het innemen van het eiwitproduct. / I consider using the protein product after the research period has ended
6. Over het algemeen ben ik tevreden met het gebruik van dit eiwitproduct. / I am satisfied with the use of the protein product
7. Ik zou dit eiwitproduct aanraden aan een ander. / I woudl recommend this protein product to others

14 vragen voor elk individueel product: ik vond de/het “product”: / 14 questions for each individual product: I thought the “product” was

- 1. Niet lekker (1) tot lekker (5) / not nice (1) to nice (5)

**Klachten**

1. Heeft u de afgelopen week na inname van de eiwitproducten last gehad van onderstaande klachten? / Did you experience dietary complaints after using the protein product during the last week?
   1. Ik heb geen klachten gehad / I did not experience complaints
   2. Misselijkheid / Nausea
   3. Braken / Vomiting
   4. Zuurbranden / Heartburn
   5. Buikpijn / Stomachache
   6. Vol gevoel / Full feeling
   7. Darmklachten / Intestinal complaints
   8. Verandering in ontlasting / Change in stool
   9. Dumping (zweten, hartkloppingen, vermoeidheid) / Dumping (sweating, palpitations, fatigue)
   10. Vastloper / Dysphagia
   11. Ik had andere klachten / I experienced other complaints

If 1. = “Ik had andere klachten” / “I experienced other complaints”

1. Welke andere klachten had u? / Which complaints did you experience?

_______________________________

If 1. ≠ “Ik heb geen klachten gehad” / “I did not experience complaints”

1. Na welke eiwitproducten kreeg u klachten? / After consuming which products did you experience complaints?
   1. Brikje drank chocolade / Brikje drink chocolate
   2. Brikje drank vanille / Brikje drink vanilla
   3. Granola bites kaneel / Granola bites cinnamon
   4. Instant drink aardbei / Instant drink strawberry
   5. Instant drink banaan / Instant drink banana
   6. Instant drink framboos / Instant drink raspberry
   7. Instant drink mango / Instant drink mango
   8. Instant drink perzik-passievrucht / Instant drink peach-passionfruit
   9. Multigranenbrood / Multigrain bread
   10. Ontbijtflakes / Breakfast cereal
   11. Reep met noten en zaden / Bar with nuts and seeds
   12. Reep yoghurt appel / Bar yogurt apple
   13. Toast natuur / Toast
   14. Wafeltjes met kaasvulling / Wafers with cheesefilling

If 1. ≠ “Ik heb geen klachten gehad” / “I did not experience complaints”

1. Hoe lang duurden de klachten na inname van het eiwitproduct gemiddeld? / How long did the complaints after using the protein product last on average?
   1. <15 minuten / <15 minutes
   2. 15-30 minuten / 15-30 minutes
   3. 30-60 minuten / 30-60 minutes
   4. >60 minuten / >60 minutes

If 1. ≠ “Ik heb geen klachten gehad” / “I did not experience complaints”

1. Kunt u de klachten na inname van het eiwitproduct verklaren door de manier van eten (te snel, teveel, te grote happen/slokken etc.)? / Can you explain the complaints after consumption of the protein product by the way of eating (too fast, too much, too large bites etc.)?
   1. Ja / Yes
   2. Nee / No

If 1. ≠ “Ik heb geen klachten gehad” / “I did not experience complaints”

1. Zijn de klachten van de eiwitproducten gedurende de week veranderd? / Did the complaints from the protein product change during the week?
   1. De klachten zijn gelijk gebleven / The complaints did not change
   2. De klachten zijn verminderd / The complaints diminished
   3. De klachten zijn verergerd / The complaints worsened

If 1. ≠ “Ik heb geen klachten gehad” / “I did not experience complaints”

1. In hoeverre denkt u dat de klachten die u had veroorzaakt worden door het eiwitproduct? / Do you think the protein product caused the complaints?
   1. Zeer onwaarschijnlijk / Highly unlikely
   2. Onwaarschijnlijk / Unlikely
   3. Niet waarschijnlijk / niet onwaarschijnlijk // Not likely / not unlikely
   4. Waarschijnlijk / Likely
   5. Zeer waarschijnlijk / Highly likely

If 1. ≠ “Ik heb geen klachten gehad” / “I did not experience complaints”

1. Zijn de klachten die u had voor u een reden om het eiwitproduct niet meer te gebruiken? / Were the complaints reason to stop using the protein product?
   1. Ja / Yes
   2. Nee / No
2. Zijn er andere voedingsmiddelen waar u regelmatig klachten van krijgt? / Are there other products in your diet that regularly cause complaints?
   1. Ja / Yes
   2. Nee / No

If 9. = “Ja”

1. Welke voedingsmiddelen zijn dit? En welke klachten krijgt u? / Which products? And which complaints do you get?

_____________________________________

If 9. = “Ja” / “Yes”

1. Zijn de klachten na inname van deze voedingsmiddelen gedurende de week veranderd? / Did the complaints from those products change during the week?
   1. Ja / Yes
   2. Nee / No
2. Heeft u naar aanleiding van het onderzoek of de vragenlijst opmerkingen of vragen? / Do you have any remarks or questions regarding the study or questionnaire?

__________________________

1. Mogen wij u benaderen met aanvullende vragen over het onderzoek of het gebruik van de app? / Can we contact you with questions about the study or the use of the mobile application?
   1. Ja / Yes
   2. Nee / No
2. Mogen wij u in de toekomst benaderen om deel te nemen aan een kort onderzoek naar het gebruik van een beweegapp? / Can we contact you in the future to ask you to take part in a study looking into the use of a fitness app
   1. Ja / Yes
   2. Nee / No
